# Rapidly Progressive Pyogenic Ventriculitis Associated With Bacterial Meningitis Caused by Streptococcus intermedius

**DOI:** 10.7759/cureus.77843

**Published:** 2025-01-22

**Authors:** Taiki Matsubayashi, Yukika Arai, Masato Obayashi

**Affiliations:** 1 Department of Neurology, National Hospital Organization Disaster Medical Center, Tokyo, JPN; 2 Department of Neurosurgery, National Hospital Organization Disaster Medical Center, Tokyo, JPN

**Keywords:** bacterial meningitis, external ventricular drainage, hydrocephalus, magnetic resonance imaging, pyogenic ventriculitis, streptococcus intermedius

## Abstract

Ventriculitis commonly arises as a complication of various central nervous system conditions. The causes of ventriculitis include both iatrogenic conditions, such as catheter-related infections, and non-iatrogenic conditions, such as community-acquired bacterial meningitis. The incidence of pyogenic ventriculitis associated with community-acquired bacterial meningitis remains unclear. Additionally, the optimal treatment strategy for pyogenic ventriculitis secondary to community-acquired bacterial meningitis remains uncertain.

A 47-year-old man presented with headache, fever, and impaired consciousness. At admission, cerebrospinal fluid analysis five days after the onset (day one) revealed elevated white blood cell count with neutrophilic predominance, increased protein levels, and significantly reduced glucose. Initial brain computed tomography (CT) showed bilateral lateral ventricular enlargement with subtle fluid accumulation in the right lateral ventricle. Despite the initiation of empirical antimicrobial therapy, follow-up CT two days later demonstrated a rapid progression of fluid accumulation in the bilateral lateral ventricles. Diffusion-weighted magnetic resonance imaging (MRI) confirmed high signal intensity within the ventricles, consistent with intraventricular pus. Emergency external ventricular drainage (EVD) was promptly performed, and cultures from the intraventricular pus identified *Streptococcus intermedius*. These findings led to a diagnosis of pyogenic ventriculitis with secondary hydrocephalus associated with bacterial meningitis. Initial clinical improvement was observed following EVD. The EVD catheter was removed 15 days after the procedure on day 18. However, the patient developed status epilepticus, necessitating a second EVD procedure on day 22. Subsequent intervention and continued antimicrobial therapy from day one until day 52 resulted in a follow-up MRI on day 55 confirming the resolution of intraventricular pus. The ventricular drain was safely removed, and the patient was discharged on day 68 in stable condition with only mild residual cognitive impairment.

This case underscores several critical considerations in managing pyogenic ventriculitis with hydrocephalus secondary to bacterial meningitis. First, careful imaging follow-up is crucial for monitoring disease progression and guiding timely interventions. Second, the prompt implementation of EVD can play a pivotal role in improving patient outcomes. Finally, EVD should be maintained until imaging confirms the complete resolution of intraventricular pus. Adhering to these management principles likely helps optimize prognosis in these challenging cases.

## Introduction

Ventriculitis is an infection of the ependymal lining of the cerebral ventricles. It frequently arises as a complication of various central nervous system (CNS) conditions, including bacterial meningitis, head trauma, intraventricular rupture of a cerebral abscess, catheter-related infections, and cerebral hemorrhage [1]. Ventriculitis secondary to bacterial meningitis, commonly referred to as pyogenic ventriculitis, can be fatal if not diagnosed and treated promptly. While the management of healthcare-associated ventriculitis is addressed in clinical practice guidelines [2], the optimal treatment strategy for pyogenic ventriculitis secondary to community-acquired bacterial meningitis remains uncertain.

We present a case of pyogenic ventriculitis secondary to community-acquired bacterial meningitis caused by *Streptococcus intermedius* (*S. intermedius*). Brain imaging confirmed the diagnosis of pyogenic ventriculitis, which rapidly deteriorated within two days despite antimicrobial therapy, necessitating emergency external ventricular drainage (EVD). This timely intervention led to a favorable clinical outcome.

## Case presentation

A 47-year-old man presented with a five-day history of persistent headache and a two-day history of fever. He was brought to our hospital due to repeated vomiting and impaired consciousness. His medical history included hypertension, untreated type 2 diabetes mellitus, and hyperuricemia. On arrival, his vital signs were as follows: body temperature, 37.5°C; blood pressure, 237/128 mmHg; heart rate, 126 beats/minute; oxygen saturation, 93% (under 6 L oxygen administration). Neurological examination revealed impaired consciousness, with a Glasgow Coma Scale (GCS) score of E3V2M5. The pupils were equal at 3 mm bilaterally, with a positive light reflex. No obvious motor paralysis was observed, but neck rigidity was noted.

Initial laboratory results are summarized in Table 1. Key serum findings included renal dysfunction, hyperglycemia with elevated glycated hemoglobin (HbA1c) level of 7.5%, and significantly elevated inflammatory markers, including a markedly increased white blood cell (WBC) count with neutrophil predominance and elevated C-reactive protein (CRP) levels. Cerebrospinal fluid (CSF) examination demonstrated elevated WBC count with neutrophil predominance, increased protein levels, and decreased glucose (CSF/serum ratio: 0.1).

**Table 1:** Laboratory parameters analyzed in the serum and CSF. HbA1c: glycated hemoglobin; CSF: cerebral spinal fluid; HIV: human immunodeficiency virus.

	Laboratory parameters	Value (units)	Reference value
Serum	White blood cells	31,200/μL	4,000-10,000
	Neutrophils	94.5%	40-70
	C-reactive protein	23.9 mg/dL	<0.5
	Blood urea nitrogen	37.7 mg/dL	8-20
	Creatinine	2.01 mg/dL	0.65-1.07
	Glucose	344 mg/dL	73-109
	Treponema pallidum antibody	Negative	Negative
	HIV-1 and HIV-2 antibody	Negative	Negative
	Interferon-gamma release assay	Negative	Negative
	HbA1c	7.5%	4.9-6.0
CSF	Color	Cloudy white	-
	CSF pressure	>350 mmH20	70-180
	White blood cells	11,184/3 μL	0-15
	Polymorphonuclear leukocyte	74%	0
	Protein	362.7 mg/dL	15-45
	Glucose	35 mg/dL	40-70

**Figure 1:**
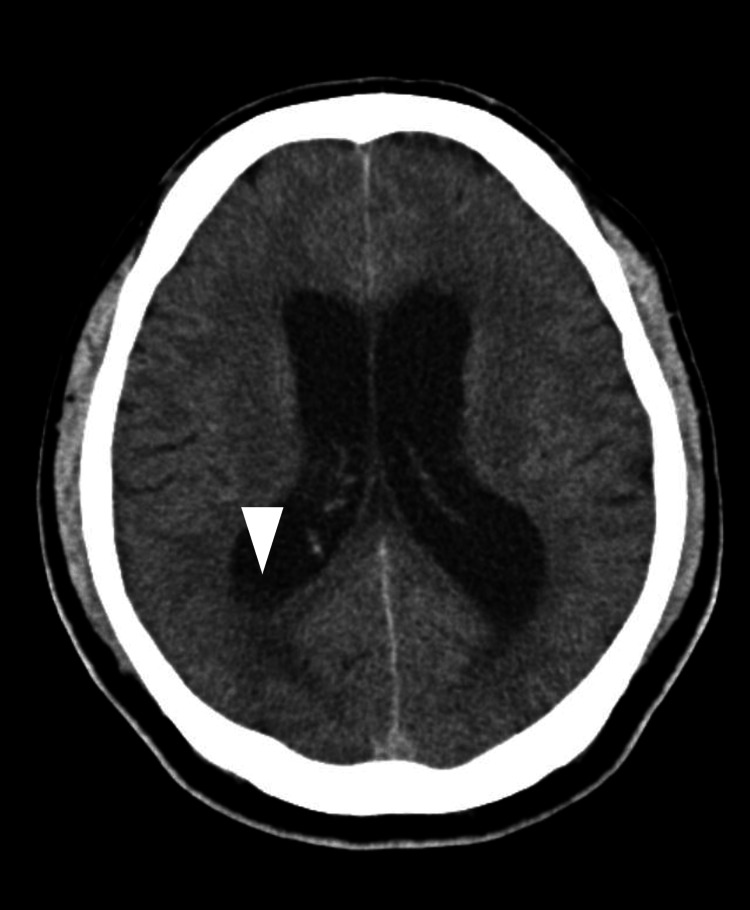
Brain CT at admission. The initial CT scan demonstrated enlargement of the bilateral lateral ventricles with subtle fluid accumulation in the right lateral ventricle (white arrowhead).

Initial brain computed tomography (CT) showed enlargement of the bilateral lateral ventricles with subtle fluid accumulation in the right lateral ventricle (Figure 1). A whole-body CT revealed no findings indicative of an abscess. Based on these findings, bacterial meningitis was suspected, and empirical antimicrobial therapy with meropenem and vancomycin was initiated, along with adjunctive dexamethasone.

**Figure 2:**
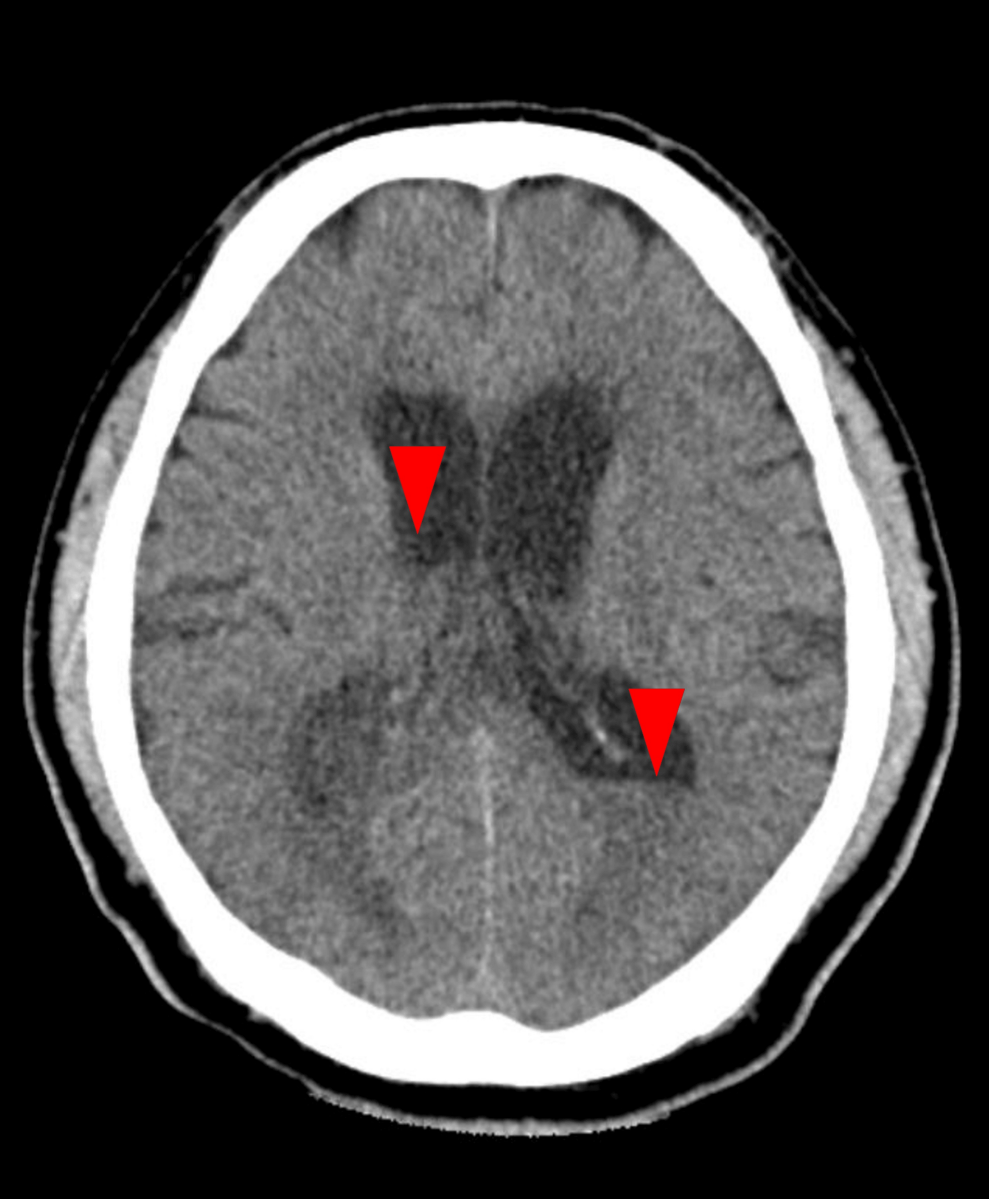
Brain CT on day three. A follow-up CT scan on day three revealed significant fluid accumulation in the bilateral lateral ventricles (red arrowheads).

Two days later on day three, his physical examination revealed a body temperature of 39.0°C, indicating persistent fever. Additionally, the level of consciousness had deteriorated to a GCS score of E1V2M5, compared to the admission findings. A follow-up CT revealed a rapid progression of fluid accumulation in the bilateral lateral ventricles compared to the initial imaging (Figure 2).

**Figure 3:**
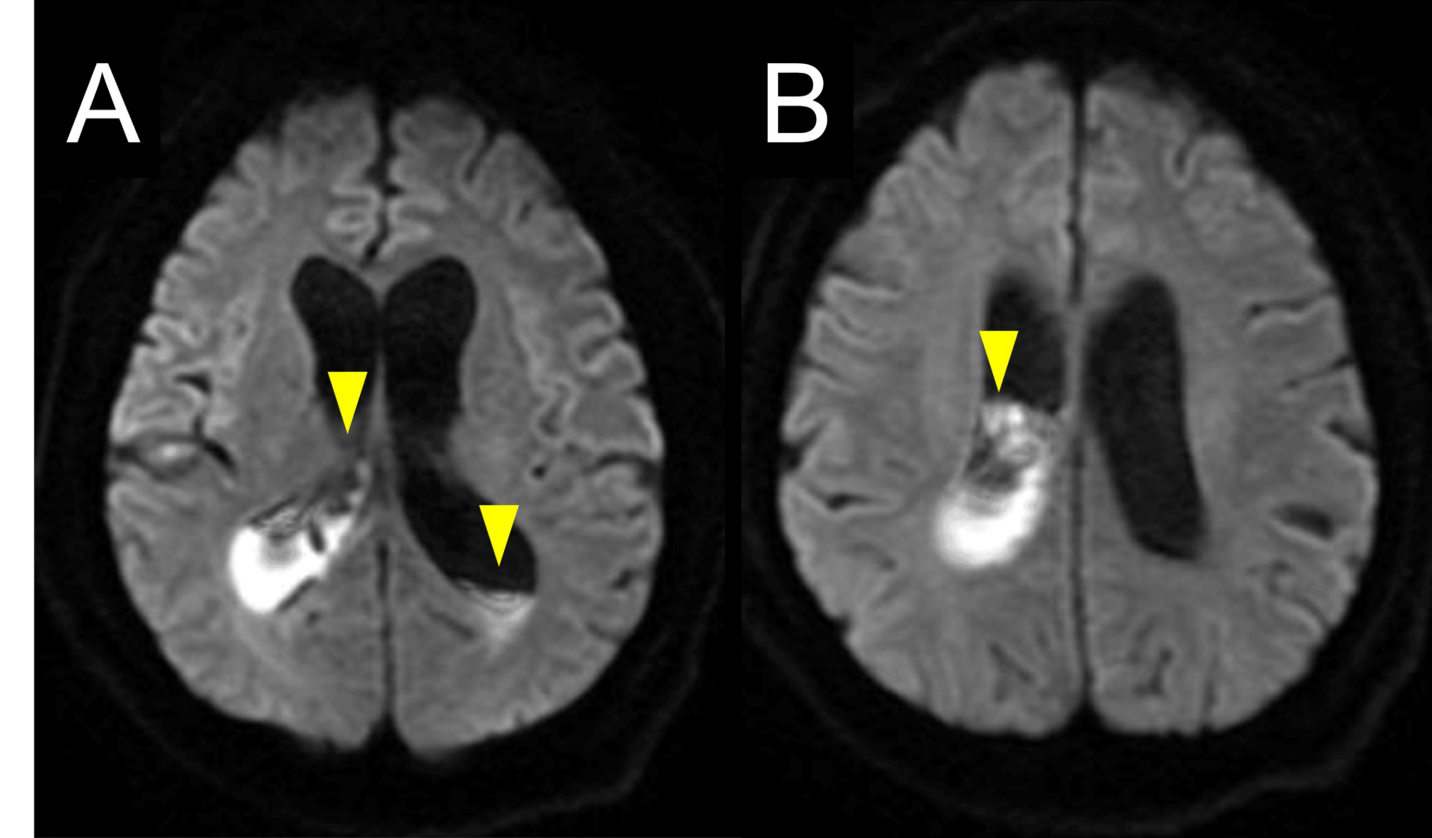
Brain MRI on day three. Diffusion-weighted imaging on day three showed high signal intensity in the lateral ventricles, consistent with intraventricular pus (yellow arrowheads).

Diffusion-weighted magnetic resonance imaging (MRI) of the brain demonstrated high signal intensity in the lateral ventricles, consistent with intraventricular pus (Figure 3). Emergency EVD was performed via a right frontotemporal puncture. Following the insertion of the external ventricular drain during surgery, pus drainage was observed to be satisfactory, with no findings suggestive of adhesions. Although blood and CSF cultures obtained on admission were negative, *S. intermedius* was isolated from the intraventricular pus culture. The cultured *S. intermedius* exhibited good sensitivity to β-lactam antibiotics. No fungi were detected in blood, CSF, or intraventricular pus culture. The patient was diagnosed with pyogenic ventriculitis and secondary hydrocephalus associated with bacterial meningitis. An examination by dentistry and oral surgeon showed no evidence of dental caries, periodontitis, or gingivitis.

**Figure 4:**
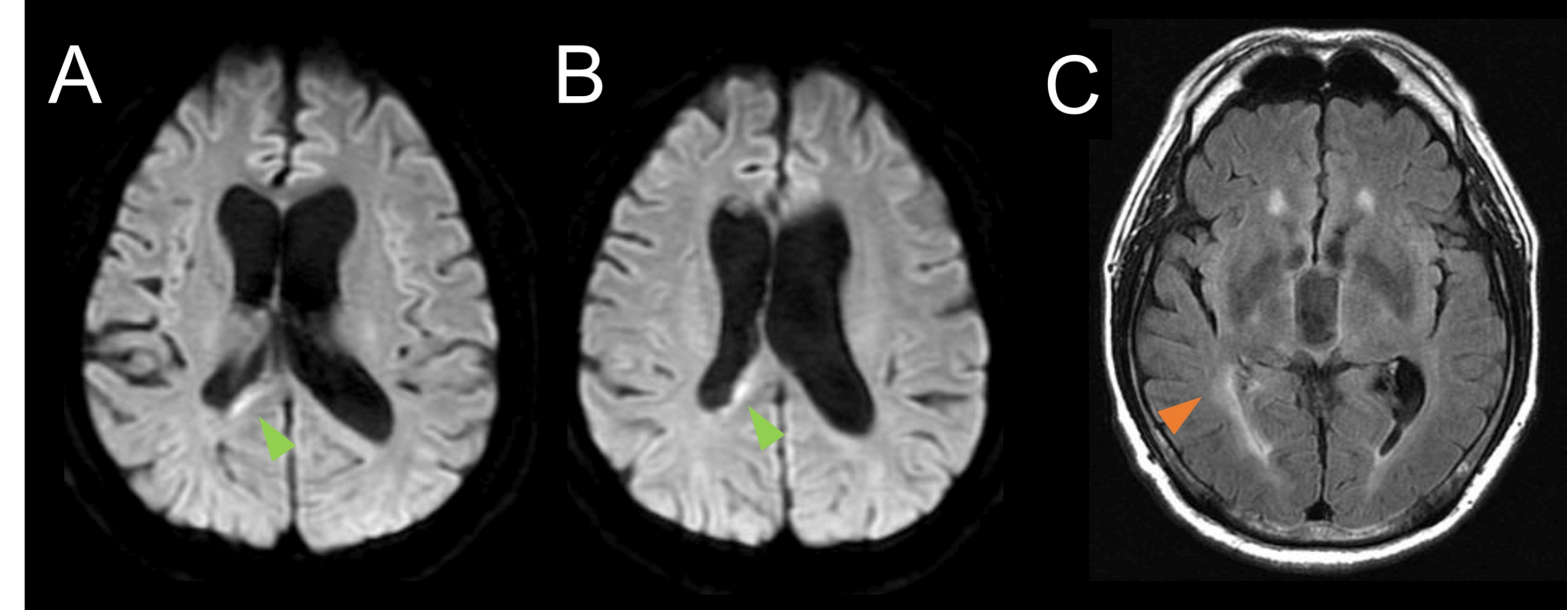
Brain MRI on day 93. Follow-up diffusion-weighted imaging on day 93 confirmed the resolution of intraventricular pus. However, enlargement of the lateral ventricles and a slightly high signal around the right lateral ventricle persisted (green arrowheads). The fluid-attenuated inversion recovery sequence revealed a narrowing of the inferior horn of the right lateral ventricle, suggestive of adhesions (orange arrowhead).

The patient’s symptoms, including fever, headache, and disorientation, showed significant improvement following EVD. A brain CT scan on day 17 revealed slight fluid retention in the right ventricle. However, given the improvement in his overall condition, the EVD catheter was removed on day 18. On day 22, the patient developed focal seizures involving the left upper and lower extremities, which progressed to sustained generalized convulsions. Status epilepticus was managed with intravenous levetiracetam, and a new EVD procedure was performed via a right frontotemporal puncture. Following the insertion of the external ventricular drain during surgery, pus drainage was satisfactory, and no evidence of adhesions was observed. Following this intervention, perampanel was initiated and continued as part of the seizure management protocol. Antimicrobial therapy with meropenem and vancomycin was administered until days 10 and 21, respectively. Subsequently, cefepime was initiated due to the presence of the EVD catheter and the need for adequate cerebrospinal fluid penetration, and it was continued until day 52. A follow-up MRI on day 55 confirmed the resolution of intraventricular pus, allowing the ventricular drain to be removed on day 56. CSF analysis on day 45 revealed normal WBC counts (11/3 µL) and protein levels (34.2 mg/dL). Additionally, blood tests on day 52 showed significant improvement in inflammation, with a WBC count of 7,800/μL and a CRP level of 0.31 mg/dL. The patient exhibited clear consciousness with a GCS score of E4V5M6 and mild residual cognitive impairment, reflected by a Mini-Mental State Examination score of 28. He was discharged home in stable condition on day 68. A follow-up MRI performed on day 93 confirmed sustained resolution of intraventricular pus (Figure 4A). Enlargement of the lateral ventricles, narrowing of the inferior horn of the right lateral ventricle suggestive of adhesions, and a slightly high signal intensity around the right lateral ventricle were observed, consistent with previous MRI findings (Figures 4B, 4C).

The clinical summary of this case is summarized in Table 2.

**Table 2:** Summary of clinical time course in this case. EVD: external ventricular drainage; GCS: Glasgow Coma Scale; MMSE: Mini-Mental State Examination.

Time course	Clinical symptom	Imaging	Imaging findings	Neurosurgical intervention
Five days before admission	Headache			
Two days before admission	Fever			
Day 1 (admission)	Impaired consciousness (GCS score: E3V2M5)	CT	Hydrocephalus, subtle fluid accumulation in the right lateral ventricle	
Day 3	Fever, impaired consciousness (GCS score: E1V2M5)	CT, MRI	Hydrocephalus, rapidly progressive pyogenic ventriculitis	First EVD
Day 10	Resolution of fever, clear consciousness (GCS score: E4V5M6)			
Day 17		CT	Hydrocephalus, subtle fluid accumulation in the right lateral ventricle	
Day 18				Removed EVD catheter
Day 22	Status epilepticus			Second EVD
Day 38	Clear consciousness (GCS score: E4V5M6)			
Day 44	Mild cognitive impairment (MMSE score: 28)			
Day 55		MRI	Adhesion, hydrocephalus, periventricular inflammation, resolution of intraventricular pus	
Day 56				Removed EVD catheter
Day 68 (discharged home)				
Day 93		MRI	Adhesion, hydrocephalus, periventricular inflammation, resolution of intraventricular pus	

## Discussion

The most notable feature of this case was the rapid progression of pyogenic ventriculitis within only two days after admission. The initial brain CT had already revealed hydrocephalus, despite only mild fluid accumulation, prompting timely follow-up imaging. In this case, intraventricular pus likely obstructed CSF circulation, leading to hydrocephalus. Additionally, stasis of CSF flow may have exacerbated inflammation within the ventricles, further promoting pus accumulation and accelerating the progression of ventriculitis.

A previous study with 98 cases of pyogenic ventriculitis identified *Streptococcus pneumoniae* as the most common causative organism [3]. However, only nine cases of adult pyogenic ventriculitis caused by *S. intermedius* have been previously reported (Table 3) [4-12]. The source of infection was identified as brain or other organ abscesses in the majority of cases (6/10). However, in four cases, including the present case, the source of infection remained unidentified. *S. intermedius* is a commensal organism of the oral cavity and a known opportunistic pathogen capable of forming abscesses in the brain and liver [13]. Notably, neurosurgical interventions were performed in almost all cases (9/10). Specifically, in seven cases, including our own, neurosurgical intervention was performed for the management of hydrocephalus, while in three cases, including our own, the intervention was aimed at controlling ventriculitis. These findings highlight the frequent occurrence of severe CNS complications requiring surgical management in cases of pyogenic ventriculitis caused by *S. intermedius*. Its ability to produce cytotoxins specifically targeting human cells may contribute to its invasive potential and the severe progression observed in cases of pyogenic ventriculitis with CNS complications. Risk factors for *S. intermedius* infections include conditions such as solid tumors, diabetes mellitus, and heavy alcohol consumption [13]. Especially, a previous study has reported that individuals with type 2 diabetes and an HbA1c level of 7-8% have a 1.53-fold higher risk of developing infections requiring hospitalization, based on adjusted incidence rate ratios, compared to those without diabetes [14]. In the present case, the patient's underlying diabetes with an HbA1c level of 7.5% likely predisposed him to this severe infection.

**Table 3:** Cases of adult pyogenic ventriculitis caused by Streptococcus intermedius. CNS: central nervous system; EVD: external ventricular drainage; VP: ventriculoperitoneal.

Study	Age	Sex	Infective source of ventriculitis	CNS complications	Neurosurgical intervention	Reason for neurosurgical intervention	Prognosis
Chen et al. [4]	56	M	Unknown	Meningitis	EVD, later VP drain	Control of ventriculitis	Survived without neurological sequelae
Allonen et al. [5]	64	M	Periodontal destruction, periapical abscesses	Hydrocephalus	Ventriculostomy	Control of hydrocephalus	Survived with slight left-sided hemiparesis
Fogg et al. [6]	74	F	Brain abscess in the basal ganglia	Brain abscess	Abscess drainage, EVD, ventriculostomy	Control of brain abscess and ventriculitis	Survived with a need for rehabilitation
Daly et al. [7]	62	M	Unknown	Hydrocephalus	VP drain	Control of hydrocephalus	Survived with a need for rehabilitation
Li et al. [8]	69	F	Chronic lung abscess	Hyperdense lesions within the cyst, multiple contrast-enhanced cystic lesions with surrounding brain edema	-	-	Survived with a need for rehabilitation
Tao et al. [9]	61	M	Erector spinae muscle abscess	Hydrocephalus	VP drain	Control of hydrocephalus	Survived with mild to moderate cognitive impairment
Dandurand et al. [10]	51	M	Multiple liver abscesses, perforated diverticulitis	Brain microabscesses, hydrocephalus, periventricular cerebritis	EVD, later VP drain	Control of hydrocephalus	Improved ventriculitis, but died due to a respiratory complication
Kamar et al. [11]	36	M	Brain abscess in the splenium	Brain abscess, hydrocephalus, meningoventriculitis, uncal herniation	EVD, later ventriculostomy	Control of hydrocephalus and herniation	Survived with improved neurologic state
Vajramani et al. [12]	63	M	Unknown	Adhesion, hydrocephalus	EVD, later VP drain	Control of adhesions and hydrocephalus	Survived with left-sided homonymous hemianopia
Our case	47	M	Unknown	Adhesion, hydrocephalus, meningitis	EVD	Control of hydrocephalus and rapidly progressive ventriculitis	Survived with mild cognitive impairment

The frequency of ventricular catheter-related ventriculitis has been reported to range from 0% to 45% [1]. However, the incidence of pyogenic ventriculitis associated with community-acquired bacterial meningitis remains unclear. An observational study involving nine cases of *Streptococcus pneumoniae* meningitis reported ventriculitis as a complication in all cases [15], suggesting that the frequency of pyogenic ventriculitis in community-acquired bacterial meningitis may be higher than generally recognized. For diagnosis, brain diffusion-weighted MRI has been reported as a highly sensitive tool [16], and was particularly useful in this case for monitoring disease course. In this case, contrast-enhanced MRI could not be performed due to renal dysfunction. However, periventricular enhancements have been reported to be observed on contrast-enhanced T1-weighted images in cases of ventriculitis, suggesting the presence of ependymitis [16].

The previous study involving 98 cases of pyogenic ventriculitis reported an in-hospital mortality rate of 30.6%, highlighting its poor prognosis [3]. Key poor prognostic factors identified in the study included the following: (1) age >65 years; (2) a GCS score <13 on admission; (3) status epilepticus; (4) hydrocephalus; and (5) positive CSF culture. Furthermore, previous studies have reported that bacterial meningitis complicated by hydrocephalus carries a high mortality rate of up to 50% [17,18]. Despite presenting with all these poor prognostic factors, except advanced age, our patient achieved a favorable outcome, being discharged home with only mild cognitive impairment. Finally, the MRI revealed enlargement of the lateral ventricles, adhesions within the right lateral ventricle, and periventricular inflammation. However, these findings were deemed sequelae, as no progression was observed. The presence of hydrocephalus and the rapid progression of ventriculitis in this case necessitated early EVD, which likely played a pivotal role in the patient’s recovery. Importantly, all patients with pyogenic ventriculitis caused by *S. intermedius* who underwent neurosurgical intervention achieved favorable control of ventriculitis (Table 3), supporting the critical role of such interventions in the management of this condition. Antimicrobial therapy remains the cornerstone of pyogenic ventriculitis treatment. However, in this case, ventriculitis progressed rapidly despite antimicrobial therapy and relapsed following the removal of the EVD catheter, accompanied by status epilepticus. These observations suggest that antimicrobial therapy alone may not suffice in managing pyogenic ventriculitis complicated by hydrocephalus. This case underscores several critical aspects of managing pyogenic ventriculitis secondary to bacterial meningitis with hydrocephalus, including the importance of careful imaging follow-up to monitor disease progression, the potential for early ventricular drainage to significantly improve outcomes, and the need to confirm the complete resolution of intraventricular pus via MRI before EVD catheter removal. Implementing these measures can help optimize the prognosis in such challenging cases.

## Conclusions

We present a case of pyogenic ventriculitis with hydrocephalus secondary to bacterial meningitis caused by *S. intermedius*. The clinical course of the ventriculitis rapidly deteriorated within two days, necessitating EVD. The fulminant progression observed in this case may be attributed to the bacteriological characteristics of *S. intermedius*.

This case highlights three key considerations in the management of pyogenic ventriculitis with hydrocephalus secondary to bacterial meningitis. First, early imaging follow-up is essential for evaluating disease progression. Second, timely EVD can significantly improve patient outcomes. Third, EVD should continue until the complete resolution of intraventricular pus accumulation is confirmed through imaging, ensuring optimal disease control and prognosis.
